# Hand surgery in Norway

**DOI:** 10.1177/1753193420930592

**Published:** 2020-06-14

**Authors:** Ole Reigstad, Line Lied

**Affiliations:** 1 Hand and Microsurgery Unit, Oslo University Hospital, Oslo, Norway; 2 Hand Unit, St. Olavs Hospital, Trondheim, Norway

## Early development

**Figure 1.**
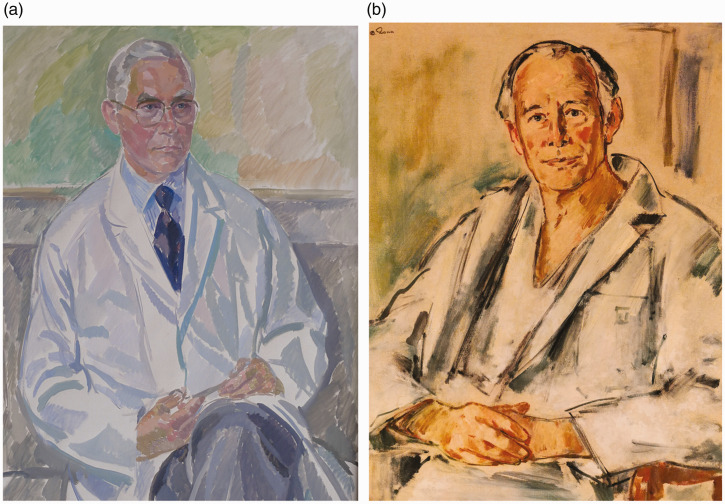
Halfdan Schjeldrup (1911–1991) and Henrich Steffens Nissen-Lie (1903–1978), Norwegian pioneers in hand surgery.

In Norway, interest in hand surgery and introduction of new methods developed from reconstructive surgery in patients who had rheumatoid arthritis or polio. After the second world war, surgeons from Norway with special interest in hand surgery, primarily the pioneers Halfdan Schjeldrup (plastic surgeon, 1911–1991, Bergen) and Henrich Steffens Nissen-Lie (orthopaedic surgeon, 1903–1978, Oslo), received their hand surgery training mainly in Great Britain and the US (Figure 1). They applied the latest concepts for treatment of nerve and tendon injuries, fractures, joint dislocations, and soft tissue injuries including burns. Both men were active nationally and internationally, sharing their experience, training students, and establishing training programmes for surgeons in Norway.

Cooperation within the Nordic countries was encouraged, and Nissen-Lie was co-founder of the Nordic Hand Club in 1951. Halfdan Schjeldrup and Henrich Steffens Nissen-Lie published numerous articles, gave lectures at the Universities of Oslo and Bergen, and received international recognition for their work, including honorary membership in the British Society for Plastic Surgery and the British Orthopaedic Association. Then and now, the common non-complicated hand operations were performed by general or orthopaedic surgeons at local hospitals throughout the country. The teaching and practice of the pioneers inspired the establishment of hand surgery units at all university clinics and at regional hospitals for the treatment of the more complex cases.

With the advancement of loupes, operating microscopes, and fine suture materials, advanced nerve surgery, including nerve grafting, was introduced and established in the 1960s and 1970s. The first replantation surgery was performed in 1983 by Astor Reigstad and colleagues, who later also introduced advanced reconstructive microsurgery, including toe transfer and free flap surgery (Hetland et al., 1985; Reigstad et al., 1992). During the 1980s and 1990s, microvascular tissue transfer became commonplace at all major hand surgery departments.

## The Norwegian Society for Surgery of the Hand

**Figure 2.**
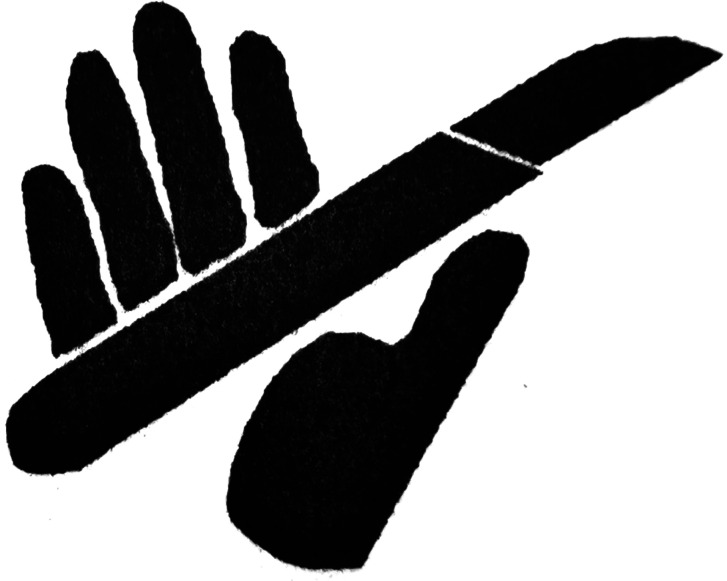
Emblem of the Norwegian Society for Surgery of the Hand.

**Figure 3.**
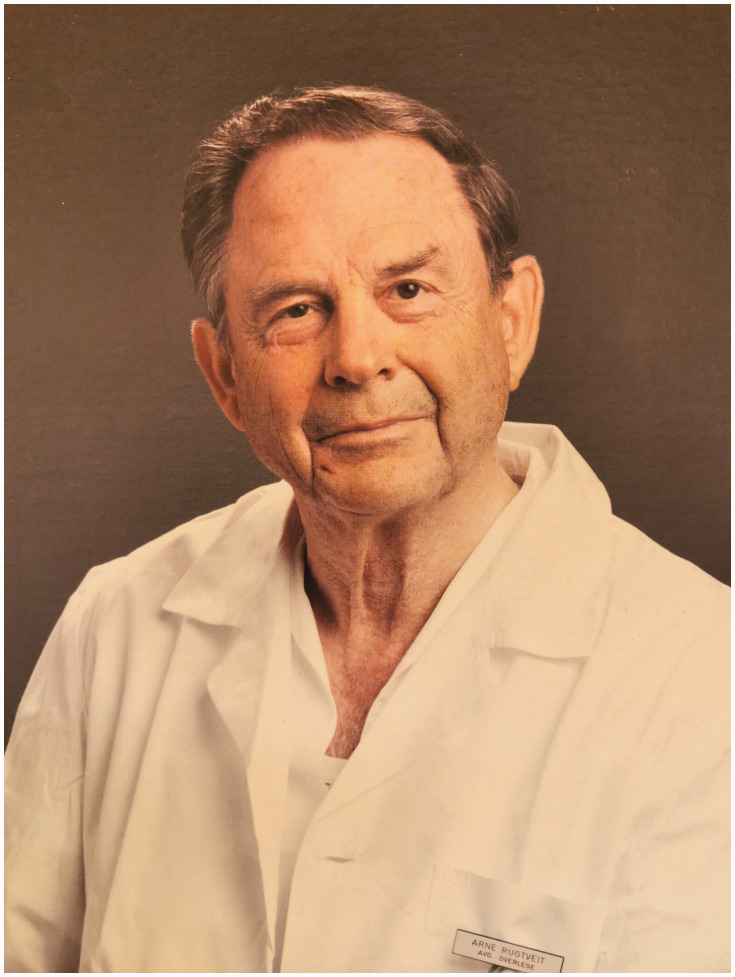
Arne Rugtveit (1922–2014), first President of the Society.

The Nordic Hand Club was founded in Copenhagen in 1951. Erik Moberg from Gothenburg, Sweden, was the first president. In the following years, the Nordic Hand Club had separate sessions during the Nordic Surgical Meeting. The first separate meeting for hand surgeons was arranged at Moberg’s department in Gothenburg in 1956. At the first Scandinavian congress in Norway in 1977, the name of the club was changed to Scandinavian Society for Surgery of the Hand (SSSH) (Boeckstyns et al., 2020). *The Norwegian Society for Surgery of the Hand* (*Norsk forening for Håndkirurgi*) was founded in 1979 (Figure 2). Arne Rugtveit (1922–2014) was the first President of the Society, which counted 22 members at the time (Figure 3). Today, the society has 61 active members and 19 retired hand surgeons. The society has been a part of the Norwegian Orthopedic Association since 2014, and is responsible for the hand and microvascular section and holds a special hand symposium at the annual Norwegian Orthopedic Conference.

Every year the Society arranges a winter meeting close to the Olympic city of Lillehammer. A renowned international guest speaker is usually invited, together with Norwegian experts on special-focus subjects. The meeting is attended by around 40 surgeons and has become an important venue for professional and social exchange. The Society also awards two scholarships and encourages research and training among its members. The members are active in the SSSH, European, and international federations for hand surgery societies as congress presenters or course faculties, and the delegates serve in the different committees and councils.

## Training of hand surgeons

Norway is a relatively large, but scarcely populated country (5 million people) with 55 hospitals spanning more than 2000 km, from the southernmost to the northern-most hospital, and 550 km from east to west. There are a large number of hand surgery patients; Norway has a high incidence of injuries (where hand injuries account for around 50%) and disorders like Dupuytren contracture, carpal tunnel syndrome, and tenosynovitis is common. Hand surgical services are available in most hospitals and are directed by trained (minimum 6 years of training) orthopaedic specialists, while more complex hand surgery is performed at special hand units (in orthopaedic or plastic surgery departments) at the university clinics and regional centres. Hand surgery training is obligatory for orthopaedic and plastic surgery specialities, but there is no formal speciality in hand surgery in Norway. An annual 5-day postgraduate instructional course in hand surgery is organized with a mixture of lectures, case discussions, and two full afternoons of upper extremity dissection. The course is compulsory for all residents in orthopaedic surgery. In 2009, a Norwegian textbook in hand surgery (revised in 2019) was authored by the members of the Society. It is the obligatory textbook for the course in hand surgery and is used by departments performing hand surgery.

Several Norwegian surgeons have obtained speciality training in hand surgery in Sweden (where hand surgery is a speciality of its own). A Norwegian Diploma in Hand Surgery, based on the FESSH (Federation of European Societies for Surgery of the Hand) Diploma, has been created. The candidates are fully trained in either orthopaedic surgery or plastic surgery (6 years) and have at least two more years of training in hand surgery, preferably at one of the university hospitals. The applicants for the diploma must display a logbook, similar to that used for the FESSH examination. Twenty-one hand surgeons have obtained the Norwegian Diploma. Eight Norwegian hand surgeons have also successfully achieved the European Diploma in Hand Surgery (EBHS).

## Current practice

Hand surgery in Norway is organized as a regional sub-speciality. The largest Norwegian university hospitals have separate hand divisions as part of their orthopaedic or plastic surgery departments, and these are all certified as hand trauma centres by FESSH (Oslo, Bergen, Trondheim, and Tromsø). Modern principles for most wrist and hand procedures have been implemented. Arthroscopic surgery has replaced open procedures, needle fasciotomy is the main procedure for primary uncomplicated Dupuytren contracture, and displaced distal radius fractures are treated operatively, usually with anterior locking plates. Flexor tendons are sutured using multiple strands and strong suture materials allowing early active motion. Arthroplasties are offered for arthrosis of the wrist, thumb basal joint, and metacarpophalangeal and proximal interphalangeal joints as an alternative to arthrodesis or interposition. Nerve and tendon transposition and transplantation are commonplace for management of sequelae of injuries or disease.

The most advanced reconstructive surgeries have been centralized to single national centres. Replantation surgery and brachial plexus surgery (obstetric/traumatic) is available at the Hand and Microsurgery Department, Division of Orthopedic Surgery, Oslo University Hospital, and the operative treatment for restoration of hand and arm function in tetraplegic patients is available at the Department of Orthopedic Surgery, Haukeland University Hospital in Bergen. Yearly between 60–80 replantations, 40–50 plexus/sequela plexus surgeries, and 15–20 tetraplegic surgeries (in 5–10 patients) are performed for our population of 5 million inhabitants.

## Research activities

Research in hand surgery has been dominated by the university departments in the largest cities. There is a professor of hand surgery at the University of Oslo and a professor of orthopaedic surgery with special interest in hand surgery in Tromsø. Annually between five and 10 peer-reviewed hand surgery articles are published. During the last 10 years six PhD candidates in hand surgery have defended their theses on radius and metacarpal fractures, flexor tendon injuries, wrist and trapeziometacarpal joint arthrosis/replacements, and cold intolerance. Ongoing research and doctoral projects include distal radius fractures, replantation surgery, brachial plexus treatment, dysmelia, and wrist replacements.
